# Supplementary material for: Plaid‐Like Spin Splitting and Chirality of Magnon Bands in Antiferromagnetic MnTe2
Source: Adv Sci (Weinh). 2026 Jul 17:e76555. Online ahead of print. doi: 10.1002/advs.76555 (PMC13379266; doi:10.1002/advs.76555)
Supplement: Supplementary file 1 — Supporting File: advs76555‐sup‐0001‐SuppMat.pdf. [file ADVS-9999-e76555-s001.pdf]

Supplementary Information

Plaid-Like Spin Splitting and Chirality of Magnon Bands in Antiferromagnetic $MnTe_2$

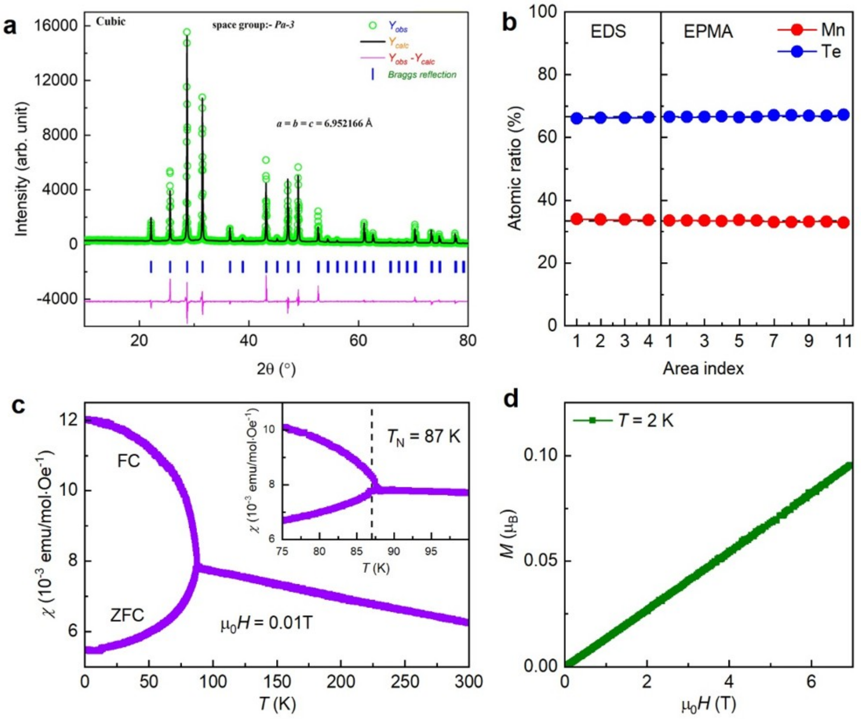

e

		Mass percent			Atomic ratio			Mn = 1	
No.	Comment	Mn	Te	Total	Mn	Te	Total	Mn	Te
No-1									
1	C1-1	17.66	81.75	99.41	33.411	66.589	100	**1**	**1.993**
2	C1-2	17.73	81.55	99.28	33.555	66.445	100	**1**	**1.980**
3	C1-3	17.83	82.17	100.00	33.510	66.490	100	**1**	**1.984**
4	C1-4	17.57	81.83	99.40	33.277	66.723	100	**1**	**2.005**
5	C1-5	17.72	81.22	98.94	33.635	66.365	100	**1**	**1.973**
No-2									
10	C2-5	16.77	84.29	101.06	31.603	68.397	100	**1**	**2.164**
11	C2-6	17.94	82.53	100.47	33.548	66.452	100	**1**	**1.981**
12	C2-7	17.64	83.45	101.09	32.923	67.077	100	**1**	**2.037**
13	C2-8	17.52	82.52	100.04	33.022	66.978	100	**1**	**2.028**
14	C2-9	17.67	82.64	100.31	33.185	66.815	100	**1**	**2.013**
15	C2-10	17.81	83.14	100.95	33.218	66.782	100	**1**	**2.010**
16	C2-11	17.56	83.19	100.75	32.893	67.107	100	**1**	**2.040**

FIG. S1. Basic characterization of the $MnTe_2$ single crystal. (a) Structural refinement via x-ray diffraction, (b) local composition measured at various spots via EDS and EPMA, (c) magnetic susceptibility, (d) magnetization up to 7 T at $T = 2$ K. In table (e) we detail the EPMA results, revealing a nearly perfect 1:2 ratio of Mn:Te.

I. BASIC CHARACTERIZATION AND SURFACE QUALITY

In this section we characterize the sample investigated in our study with respect to its structure, composition, and magnetic properties. First, we focus on structure and composition. In Fig. S1a, we show results of XRD refinement, confirming that our sample crystallizes in the cubic space group $Pa\bar{3}$ with a lattice parameter of 6.952 Å, consistent with previous reports [S1]. We also remark that there is no secondary phase detected in these measurements. Panel b summarizes the results of stoichiometry analysis obtained through EDS (energy-dispersive x-ray spectroscopy) and EPMA (electron probe microanalysis) measurements. Both techniques reveal a Mn:Te ratio of about 1:2, independent of position. In panels (c) and (d) we show results of magnetic characterization. A clear, sharp transition exists at $T_N = 87$ K – consistent with literature data [S2]. The magnetization curve, taken at 2 K, shows a gradual, continuous increase in magnetization up to a maximum field of 7 T. The table shown in panel (e) quantifies the results obtained from EPMA measurements.

A final comment related to the surface quality of the as-grown sample is warranted, based on our Raman measure-

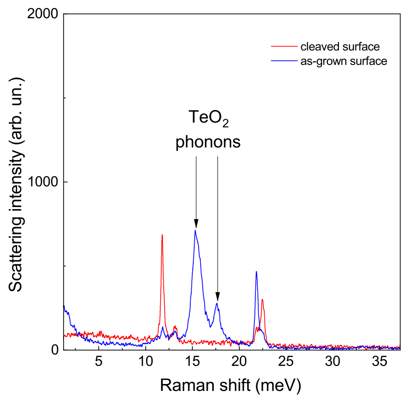

FIG. S2. Room temperature Raman spectra of $MnTe_2$ taken on the as-grown surface (blue) and on a freshly cleaved surface (red).

ments. In Supplementary Fig. S2 we show a room temperature Raman spectrum taken on the as-grown surface (blue curve). It is dominated by two broad modes in the energy range 15-20 meV. In contrast, after cleaving the sample inside the glove box and sealing it inside the cryostat without air exposure, we obtain a flat, clean background in the same energy range, together with sharp, well-defined phonon modes assigned to $MnTe_2$. These same broad modes that we observe on the as-grown surface were also reported in other Te-related compounds and are commonly assigned as TeO_2 modes [S3, S4] that arise from surface oxidation, or due to a surface layer formed in the synthesis process. In all of our subsequent Raman measurements, these defect modes are absent, suggesting that once a surface layer is removed, the sample maintains a high quality throughout the bulk.

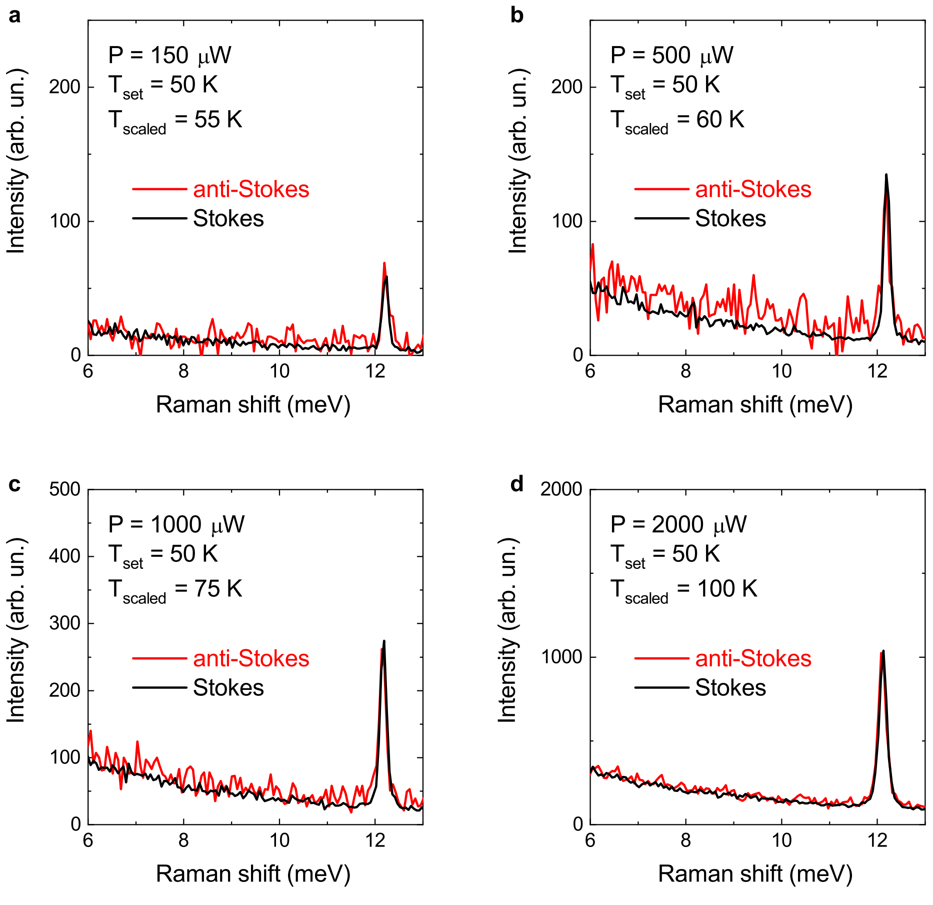

FIG. S3. Comparison of Raman spectra measured on the Stokes- (black) and anti-Stokes side (red). The sample temperature was set to 50 K. The Boltzmann factor was scaled such that an optimal overlap between both spectra was achieved. The corresponding temperature, T_{scaled} is indicated in each panel. The laser power was set to 150 μW (a), 500 μW (b), 1000 μW (c), and 2000 μW (d).

II. ESTIMATION OF THE LOCAL LASER HEATING IN THE RAMAN SCATTERING EXPERIMENT

To confirm that our data was not affected by excessive laser heating, we compare the ratio of Stokes- and anti-Stokes intensities (I_S and I_{AS}, respectively) of a phonon at 12 meV at a set sample temperature of 50 K (see Supplementary Fig. S3). The ratio of these intensities is set by $\frac{I_{AS}}{I_S} = \left(\frac{\omega_i+\omega_s}{\omega_i-\omega_s}\right)^4 \exp\left(-\frac{\hbar\omega_s}{k_B T}\right)$. Here, ω_i and ω_s denote incident and scattered frequencies, $\hbar$ is Planck's constant, k_B is the Boltzmann constant, and T is the local sample temperature. For a laser power of 150 μW we find an optimum scaling between Stokes and anti-Stokes for a local temperature of 55 K. As we increase the laser power, the local sample temperature gradually rises, and for an incident laser power of 2 mW we register a temperature increase of 50 K. Since all temperature-dependent Raman data have been conducted with a laser power of 150 μW, we correct all our data for a temperature offset of 5 K.

An additional, independent estimate of the amount of laser heating is obtained by tracing the magnetic phase transition temperature through the energy of the 2.5 meV magnon mode. As the temperature raises and $MnTe_2$ approaches T_N, the magnon energy softens towards 0 meV, following the common behavior of an order parameter. In the presence of substantial local heating effects through the incident laser, the softening to 0 meV would appear at a noticeably lower temperature than T_N. Instead, as shown in Supplementary Fig. S4, T_N closely aligns with the disappearance of the magnon frequency towards zero energy.

Having convincingly established the existence of only minor local laser-induced heating, we now focus on the observed Stokes- / anti-Stokes intensity ratio of the chiral magnon. In Supplementary Fig. S5 we plot both Stokes- (positive energies) and anti-Stokes ranges (negative energies) in the same graph, for data taken in RL (red) and LR (blue) polarization. Here, we particularly would like to focus on the blue curve, where the anti-Stokes intensity significantly dominates over the Stokes intensity. Using the Boltzmann factor to scale these two ranges yields negative

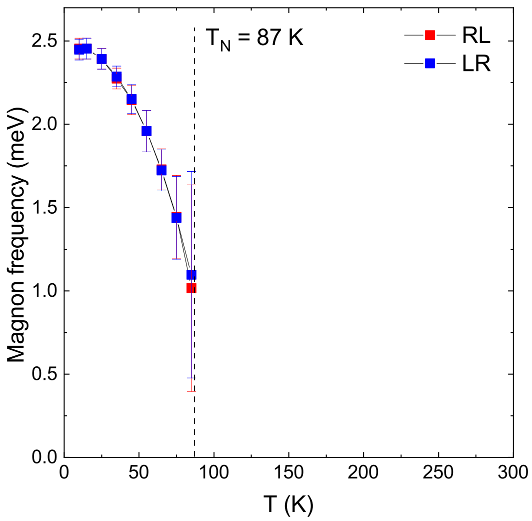

FIG. S4. Temperature-dependence of the magnon frequency extracted from RL- and LR polarization datasets (red and blue, respectively). The dashed line denotes T_N.

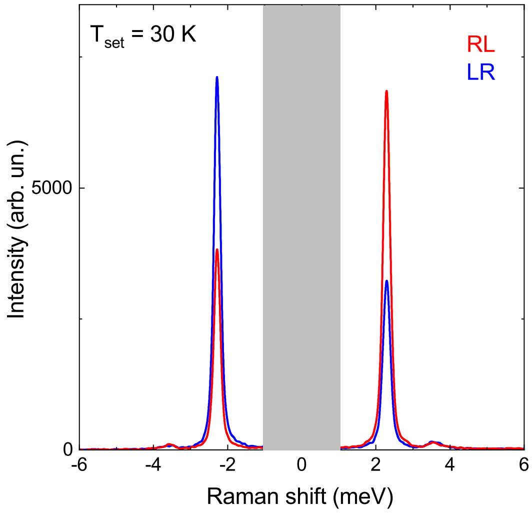

FIG. S5. Comparison of the Stokes- and anti-Stokes intensity measured at an intermediate temperature of 30 K in RL and LR configuration.

temperatures, which is physically not meaningful. Likewise, when inspecting the overall intensity on the anti-Stokes side (RL + LR), it is still larger than the overall intensity on the Stokes side.

Taken together, we can confirm that the magnon exhibits a behavior distinct from that of the phonon modes, and cannot be explained by simple thermal population effects. The most likely origin is that the excitation behaves as a chiral magnetic oscillator. Circularly polarized photons carry helicity, and in a chiral magnetic background the Raman matrix elements for magnon creation and annihilation become inequivalent. In this vein, the imbalance reflects an intrinsic violation of reciprocity between opposite helicity channels induced by magnetic chirality and broken time-reversal symmetry. Additional factors that may influence the precise imbalance include magnetic domains and possible nonreciprocal magnon dynamics.

III. POSITION DEPENDENCE OF THE MAGNON CHIRALITY

In preparation of the experiment, as-grown samples were freshly cleaved to reveal mm-sized, flat and shiny surfaces with well-defined crystal habitus (square- or rectangular-shaped facets, see Supplementary Fig. S6m). These clean, shiny facets allow to mount the samples perpendicular to the incident laser beam. Furthermore, their existence indicates that the natural cleavage plane corresponds to the [100], [010], or [001] surfaces. Additional confirmation on the proper alignment of the crystal is obtained from the observed symmetry properties of the optical phonons, which can be fitted to their respective Raman tensors (see Fig. 2, main text), confirming that we are investigating the 100, 010, or 001 surface. Any substantial misalignment of the sample that could potentially explain the low symmetry of the magnon polar plots would also be reflected in phonon symmetry anomalies, which are absent.

To further test reproducibility and position dependence, we performed measurements at ten randomly selected spots across a mm-range at two surfaces that are separate from each other but parallel to each other. As seen in Supplementary Fig. S6a-j, a clear RL/LR imbalance is present at all ten spots, which qualitatively confirms our previous results. However, the sign and the magnitude of the imbalance depend on the surface, as summarized in panels k and l, confirming the existence of magnetic domains with distinct domain-dependent chirality.

Based on these observations, we conclude that an unusual imbalance between RL and LR polarization channels, together with an unexpected Stokes / anti-Stokes ratio is consistently observed in the magnetically ordered phase of $MnTe_2$. However, the precise magnitude and sign of the asymmetry may depend on sample orientation, cooling process, and magnetic domain configuration, collectively evidencing an intrinsic magnetic origin of the observed chiral Raman response in $MnTe_2$.

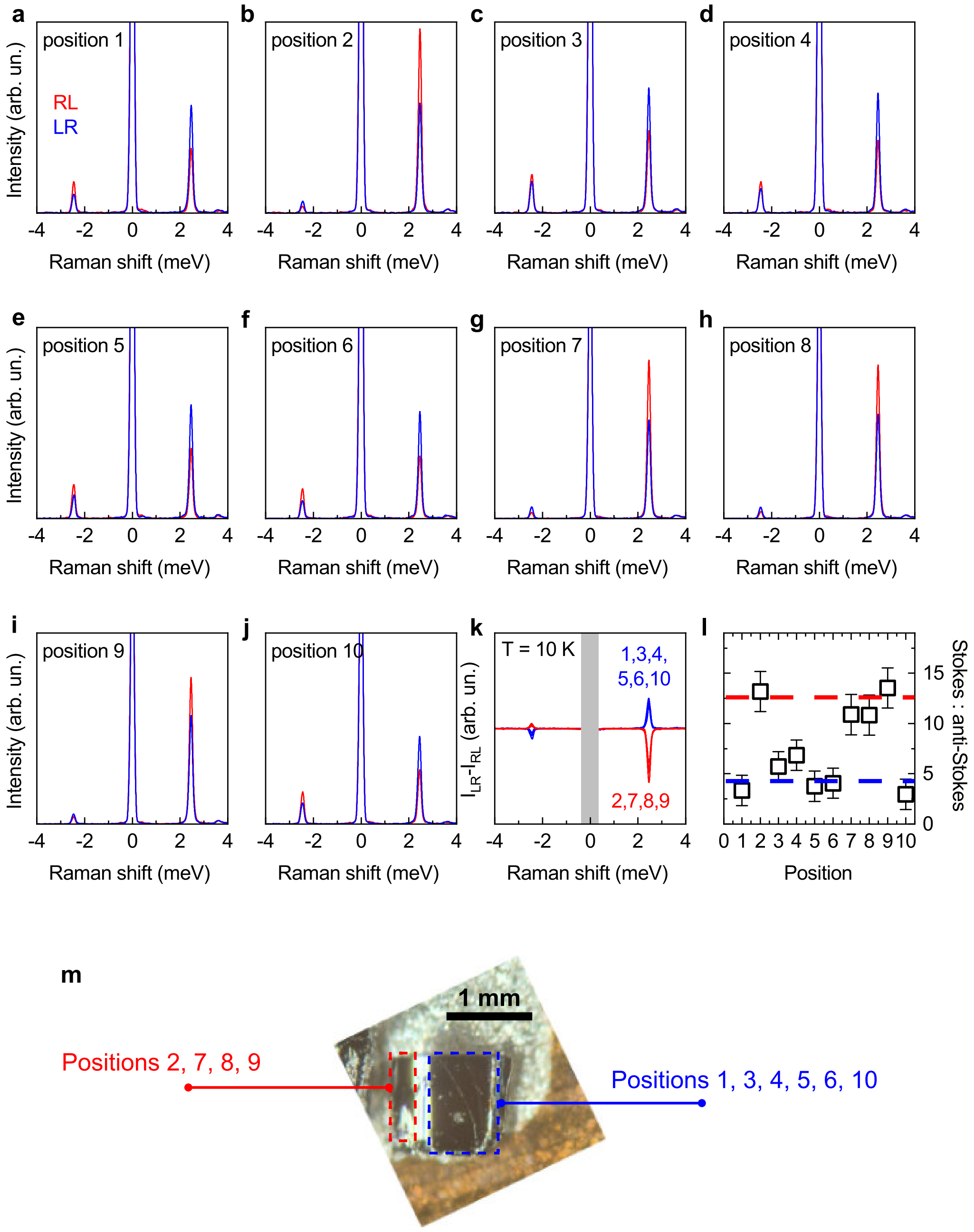

FIG. S6. (a)-(j) Raman spectra measured at ten different positions across both surfaces at a temperature $T = 10$ K, revealing two nearly opposite responses in intensity imbalance between Stokes and anti-Stokes. (k) Intensity difference $I_{LR} - I_{RL}$ calculated for each of the ten positions with two distinct results marked in blue and red. (l) Ratio of Stokes intensity to anti-Stokes intensity obtained for the ten independent positions, emphasizing the two distinct regimes. (m) Optical image of a freshly cleaved $MnTe_2$ single crystal showing two parallel surfaces (marked by dashed blue and dashed red rectangles).

IV. RAMAN SPECTRA

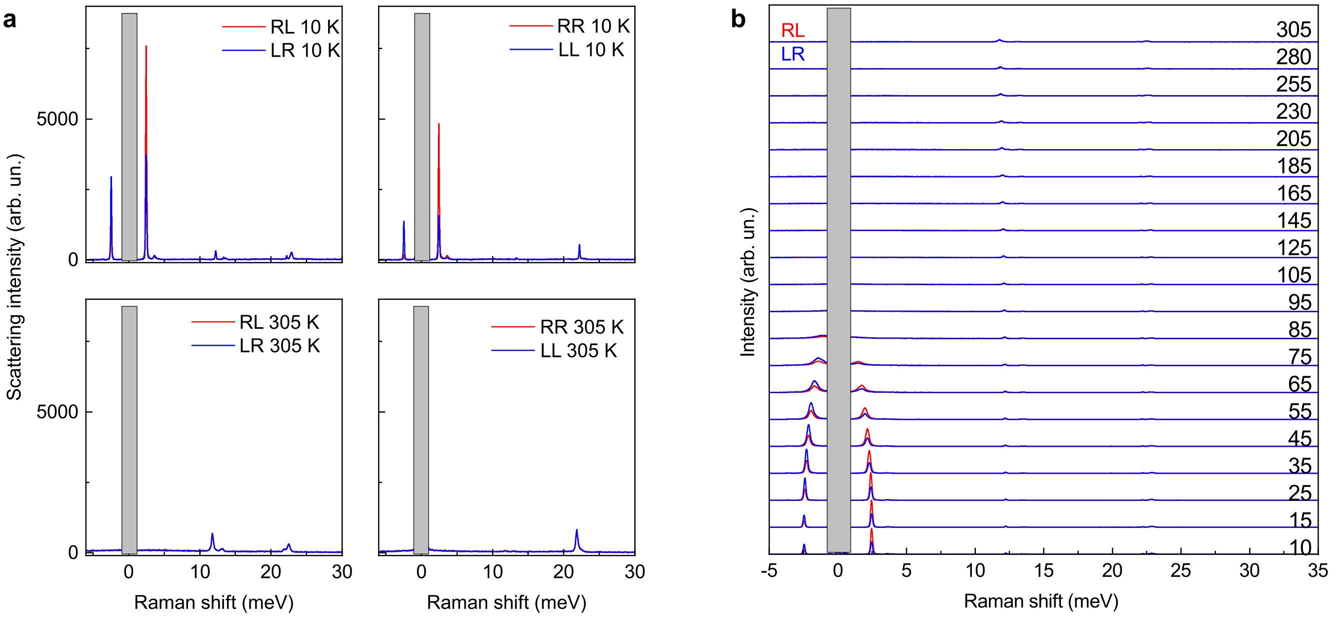

FIG. S7. (a) Direct comparison of Raman spectra collected in cross-circular polarization (RL = red; LR = blue) and co-circular (RR = red; LL = blue) in the magnetically ordered phase (T = 10 K) and above the ordered phase (T = 305 K). (b) Temperature evolution of Raman-active excitations probed in RL polarization (red) and in LR polarization (blue). Respective temperatures in units of K are indicated on the right hand side.

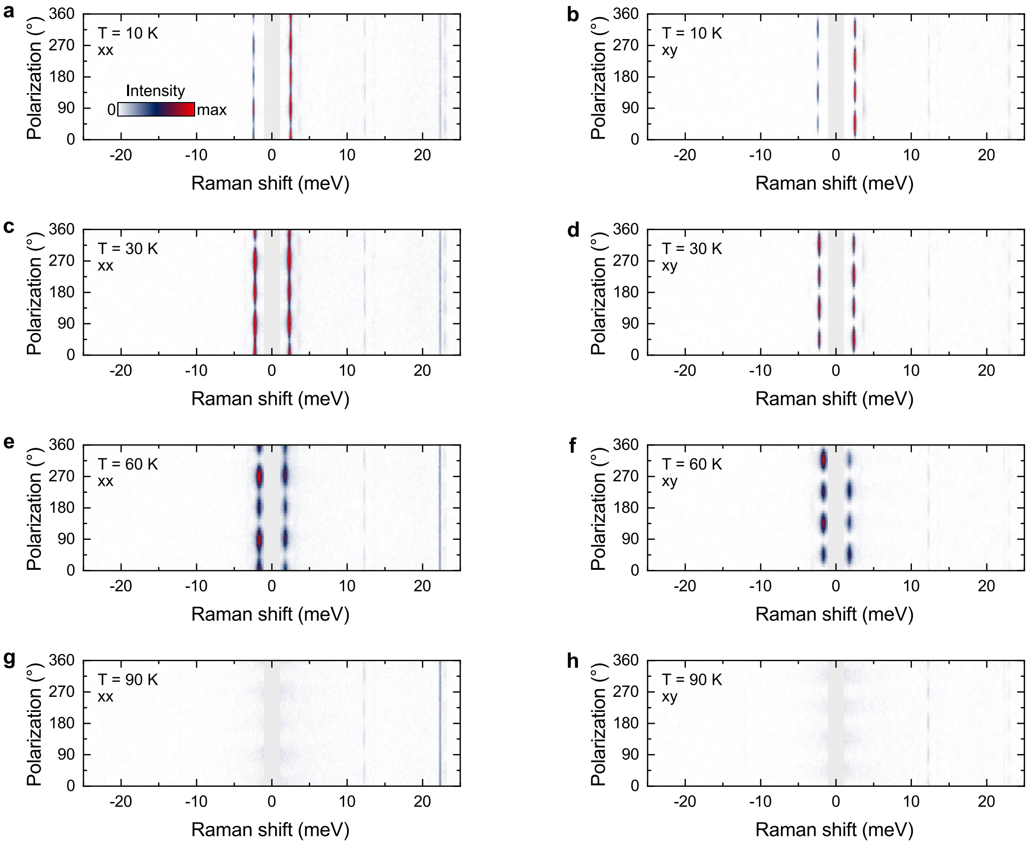

FIG. S8. In-plane polarization dependence measured at four different temperatures, 10 K, 30 K, 60 K, and 90 K. The left column shows results obtained in parallel (xx) configuration; the right column shows results obtained in crossed (xy) polarization. Note that both Stokes- (positive energies) and anti-Stokes (negative energies) ranges are displayed.

V. TEMPERATURE EVOLUTION OF THE MAGNON'S IN-PLANE ANISOTROPY

In the main text, the in-plane anisotropy of magnons is quantified at a base temperature of 10 K. A full dataset measured at temperatures of 10 K, 30 K, 60 K, and 90 K (i.e., slightly above T_N) shows the evolution of anisotropy as magnetic order gradually diminishes (see Supplementary Fig. S8). We quantify the anisotropy by extracting intensity linecuts of the 2.5 meV magnon branch measured in parallel (Supplementary Fig. S9) and in crossed (Supplementary Fig. S10) polarization on the Stokes-side and on the anti-Stokes side as a function of temperature, establishing its correlation with magnetic ordering. To quantify the angular anisotropy of the low-energy magnetic excitations, we apply a phenomenological four-component fit to the experimental data, accounting for a four-fold rotational symmetry with additional two-fold intensity modulation. The fitted function is given as $I(\theta) = |b + (a - b)\cos^2\theta|^2 + |d + (c - d)\sin^2\theta|^2$. Here, θ denotes the angle between polarization of the incident light and the crystallographic a axis, and a, b, c, d are fitting constants related to the measured intensity.

The resulting fit parameters are summarized in Table S1, together with the anisotropy parameter, that quantifies the intensity ratio of the two lobes. As shown in Supplementary Figs. S9 and S10, the low-temperature response is dominated by a pronounced two-fold anisotropy superimposed on the underlying four-fold rotational symmetry. Upon heating the sample towards T_N, the amplitude of the two-fold component decreases continuously, while the four-fold contribution becomes progressively dominant. Above T_N, at 90 K, the gapped magnon excitation is replaced by gapless spin fluctuations (quasi-elastic scattering) with a nearly four-fold symmetry profile. A weak remnant two-fold character suggests an incipient phase transition to a lowered magnetic space group. To further quantify the chirality-related asymmetry, we plot the ratio between the large and small lobes of the fitted polar profiles as a function of temperature in Supplementary Fig. S11.

Although the statistics is somewhat limited, all analyzed datasets (xx and xy geometries, Stokes and anti-Stokes channels) consistently exhibit the same systematic trend: the anisotropy continuously relaxes toward a fourfold-

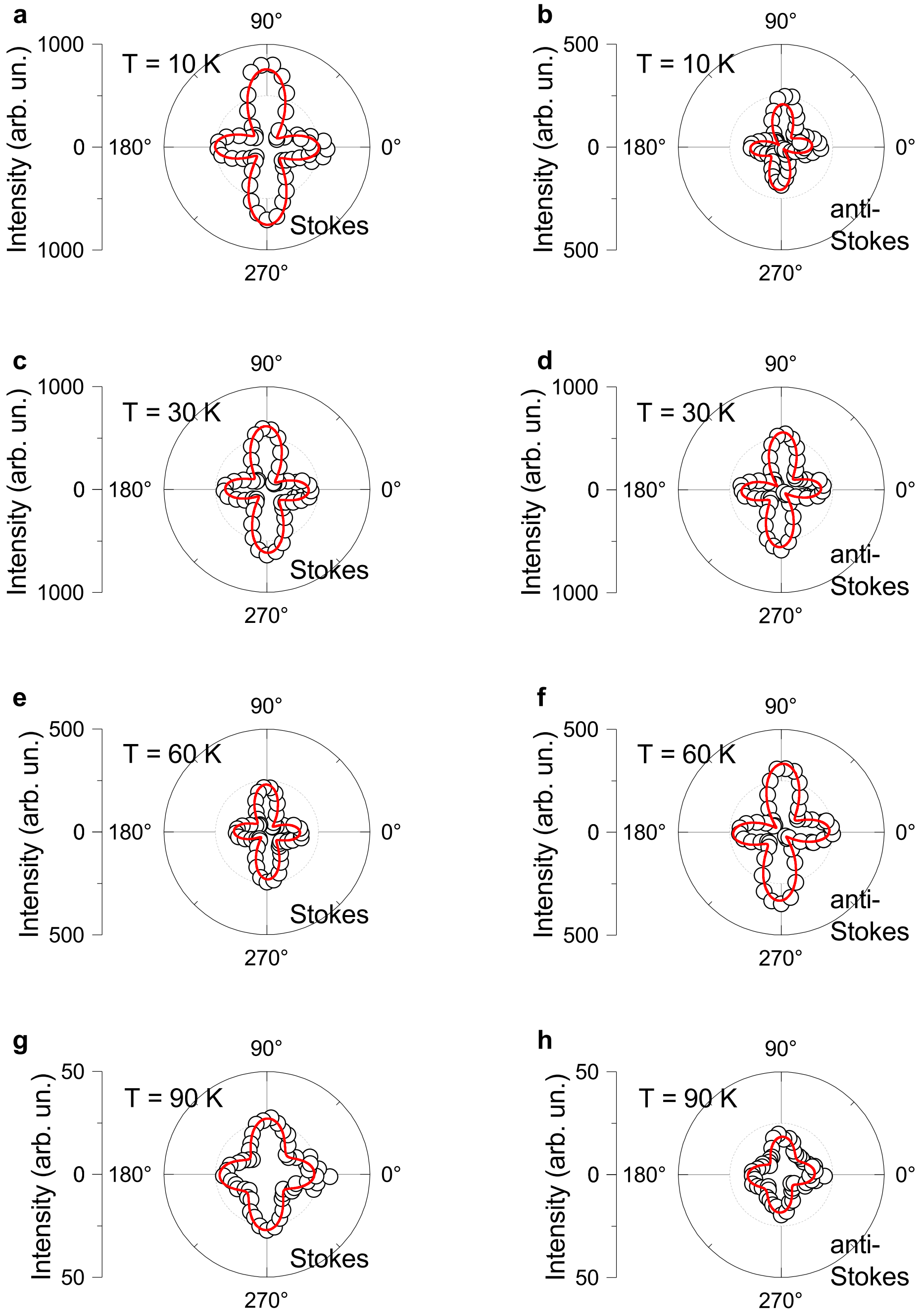

FIG. S9. Polar plots of the 2.5 meV excitation intensity measured on the Stokes-side and on the anti-Stokes side in parallel (xx) configuration at four different temperatures, 10 K, 30 K, 60 K, and 90 K. Black circles represent experimental data and red lines show fits using the four-component phenomenological model.

symmetric response, corresponding to a lobe ratio approaching 1:1 as magnetic order weakens. Importantly, this clear trend supports the robustness of the extracted symmetry evolution and its intimate connection to the magnetic ordered state.

As this detailed analysis yields important insights into the symmetry properties of the 2.5 meV magnon branch, a similar analysis would be desirable for the higher-lying magnon at 3.6 meV. Unfortunately, its overall weak intensity at low temperatures, which is further diminished towards intermediate temperatures, restricts us from extracting any reliable, meaningful linecuts with sufficient signal-to-noise ratio. For this reason, we restrict the quantitative symmetry analysis to the stronger and more robust 2.5 meV mode.

In addition to the above characterization of chirality, we also provide a temperature-dependent analysis of the the

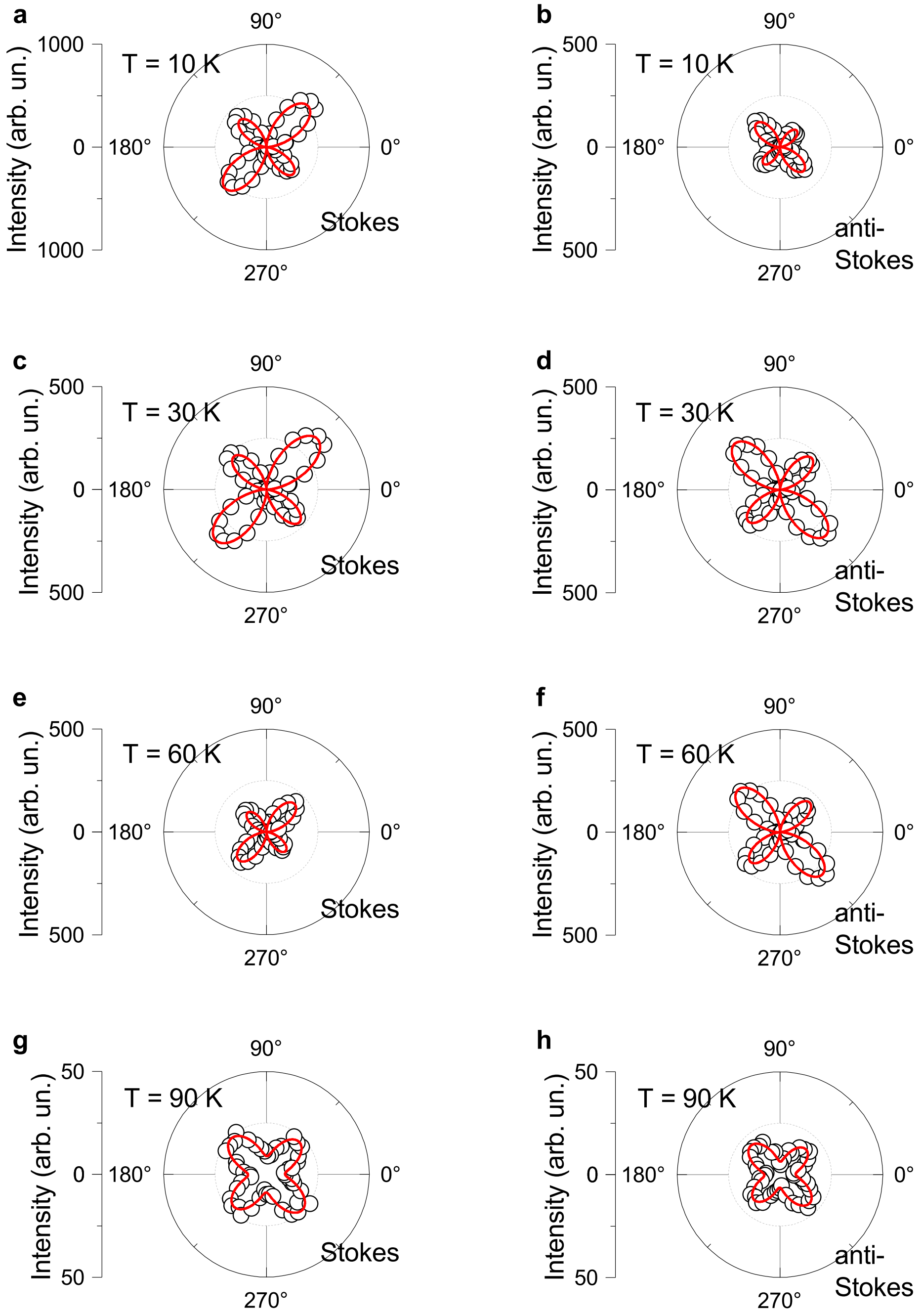

FIG. S10. Polar plots of the 2.5 meV excitation intensity measured on the Stokes-side and on the anti-Stokes side in crossed (xy) configuration at four different temperatures, 10 K, 30 K, 60 K, and 90 K. Black circles represent experimental data and red lines show fits using the four-component phenomenological model.

spectral weight of the intensity difference between RL and LR channels for the Stokes- and anti-Stokes intensities of the chiral magnon at 2.5 meV, see Supplementary Fig. S12. Consistent with the color contour plot in Fig. 1f (main text) we observe that the extracted imbalance of integrated intensities $|I_{RL} - I_{LR}|$ is gradually diminished on approaching T_N, supporting the notion that the observed chirality in $MnTe_2$ is intimately associated with magnetic order. Collectively, the combination of helicity-resolved Raman spectroscopy, angle-resolved symmetry analysis, temperature-dependent evolution, and theoretical modeling provides strong evidence for chirality-dependent magnon dynamics in $MnTe_2$.

TABLE S1. Fit parameters a, b, c, d to polar plots of the magnon intensity extracted from datasets meausred in parallel (xx) and crossed (xy) polarization on the Stokes- and on the anti-Stokes side.

		a	b	c	d	Resulting ratio of lobe intensities
10 K	Stokes xx	2.89 ± 0.19	-18.83 ± 0.45	12.19 ± 0.45	12.98 ± 0.45	1.50 ± 0.10
	Stokes xy	2.90 ± 1.33	2.88 ± 1.59	2.34 ± 0.17	-18.96 ± 0.38	1.55 ± 0.10
	anti-Stokes xx	1.24 ± 0.23	-10.58 ± 0.58	6.04 ± 0.67	3.90 ± 1.14	1.69 ± 0.15
	anti-Stokes xy	1.51 ± 0.53	1.42 ± 0.68	1.05 ± 0.06	12.65 ± 0.12	1.42 ± 0.15
30 K	Stokes xx	2.72 ± 0.16	-16.53 ± 0.40	11.46 ± 0.35	12.72 ± 0.41	1.52 ± 0.10
	Stokes xy	2.79 ± 0.75	2.71 ± 0.93	1.85 ± 0.11	-14.74 ± 0.27	1.55 ± 0.10
	anti-Stokes xx	2.22 ± 0.12	-16.97 ± 0.31	9.81 ± 0.35	7.18 ± 0.55	1.44 ± 0.10
	anti-Stokes xy	2.77 ± 0.50	2.58 ± 0.64	1.50 ± 0.08	17.50 ± 0.16	1.45 ± 0.10
60 K	Stokes xx	1.47 ± 0.10	-10.49 ± 0.24	7.05 ± 0.22	7.91 ± 0.25	1.44 ± 0.15
	Stokes xy	2.54 ± 0.46	2.38 ± 0.59	1.23 ± 0.09	-11.17 ± 0.20	1.45 ± 0.15
	anti-Stokes xx	1.66 ± 0.11	-13.23 ± 0.25	7.59 ± 0.29	5.71 ± 0.45	1.42 ± 0.15
	anti-Stokes xy	2.63 ± 0.61	2.69 ± 0.71	1.40 ± 0.09	16.82 ± 0.19	1.42 ± 0.15
90 K	Stokes xx	0.29 ± 0.06	-3.27 ± 0.15	3.50 ± 0.08	3.37 ± 0.11	1.17 ± 0.20
	Stokes xy	2.98 ± 0.63	2.99 ± 0.08	-0.14 ± 0.05	-3.95 ± 0.05	1.09 ± 0.20
	anti-Stokes xx	0.19 ± 0.05	-2.67 ± 0.12	3.01 ± 0.07	2.77 ± 0.09	1.13 ± 0.20
	anti-Stokes xy	2.63 ± 0.05	2.76 ± 0.06	0.18 ± 0.04	3.61 ± 0.08	1.18 ± 0.20

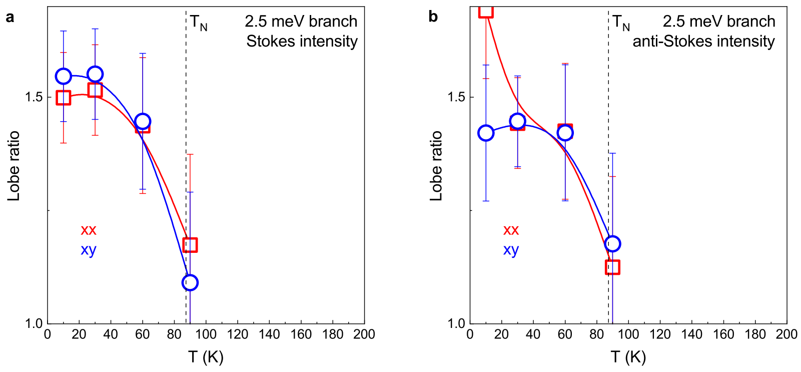

FIG. S11. Temperature dependence of the large-lobe/small-lobe intensity ratio extracted from the four-component fits to the 2.5 meV magnon response on the Stokes side (a) and the anti-Stokes side (b).

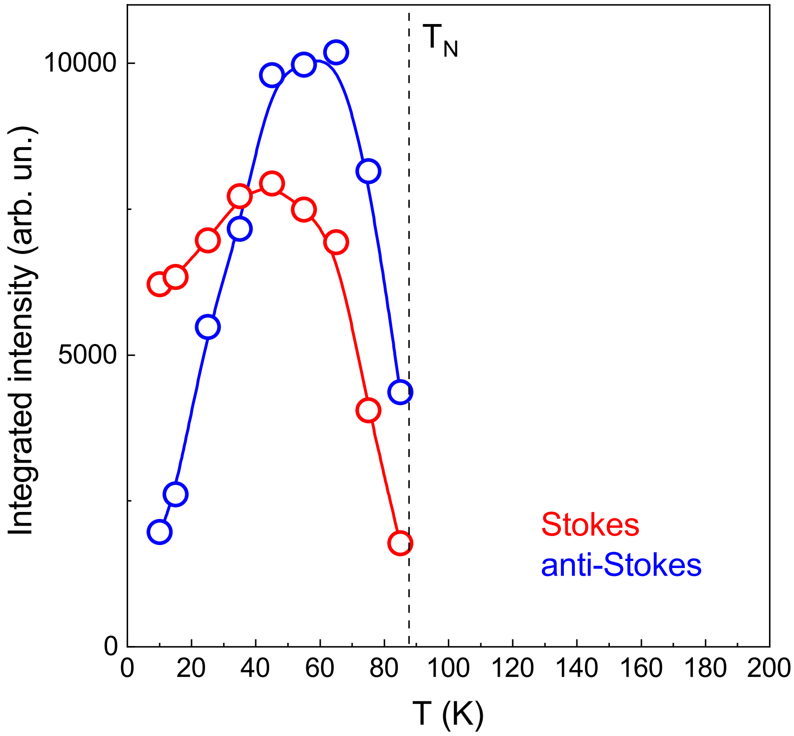

FIG. S12. Integrated intensity of the quantity $|I_{RL} - I_{LR}|$ for the 2.5 meV magnon as a function of temperature. Red symbols denote the Stokes region, while blue symbols were extracted from the anti-Stokes side.

VI. THIRD- AND FOURTH-NEIGHBOR COUPLINGS

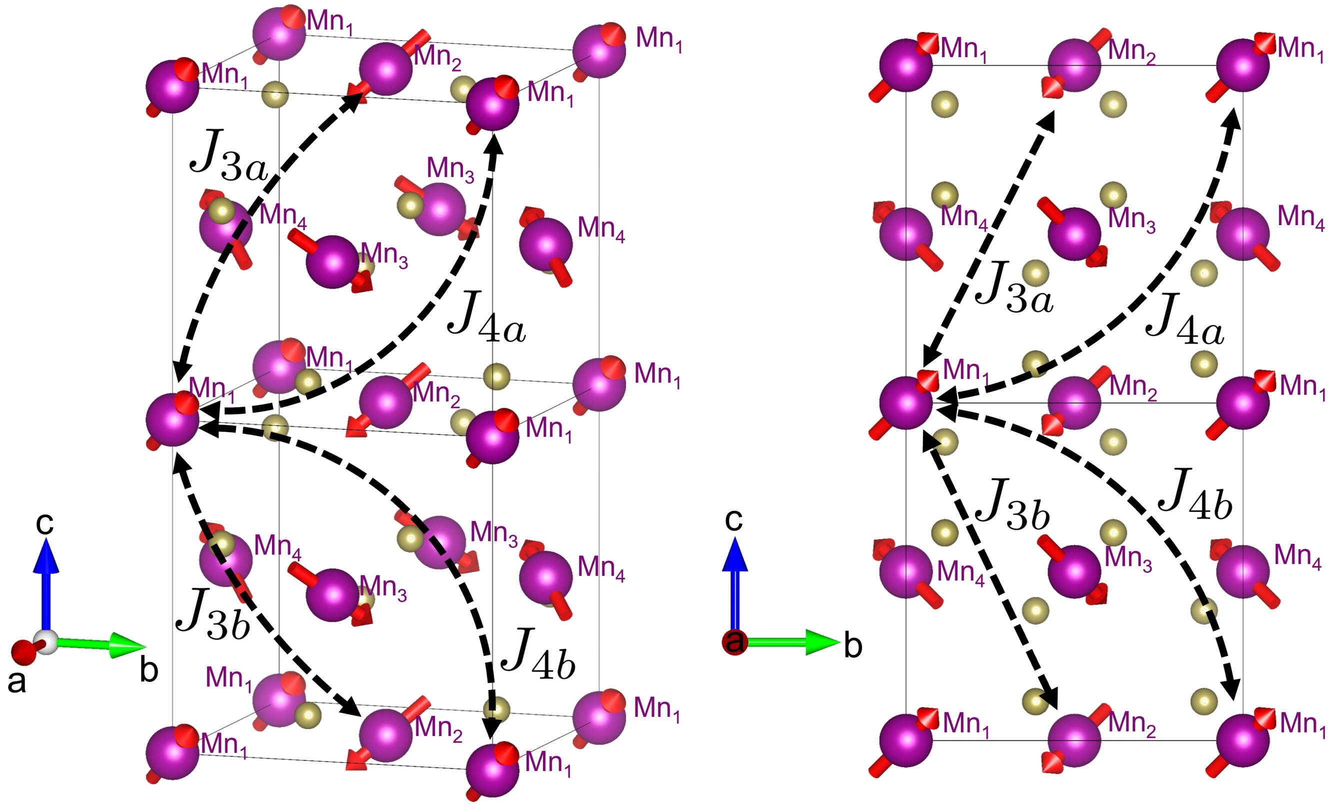

FIG. S13. Representative magnetic interaction of third and the fourth nearest-neighbor Mn pairs. Mn_1–Mn_4 label the four magnetically distinct Mn sublattices, and the red arrows indicate the spin orientations on each Mn site. The corresponding calculated magnetic interaction parameters are summarized in Table S2.

	J_n	D_n^x	D_n^y	D_n^z	Γ_n^{xx}	Γ_n^{yy}	Γ_n^{zz}	Γ_n^{xy}	Γ_n^{yz}	Γ_n^{zx}
$n = 3a$	−0.003	−0.014	0.005	0.000	0.000	−0.002	0.002	0.000	0.002	0.000
$n = 3b$	0.040	0.008	0.000	−0.002	−0.002	0.002	0.000	−0.002	−0.002	−0.002
$n = 4a$	−0.005	0.000	0.000	0.000	0.000	0.000	0.000	0.002	0.000	0.000
$n = 4b$	0.059	0.000	0.000	0.000	−0.002	0.000	0.002	−0.002	0.000	−0.002

TABLE S2. The calculation results of magnetic interactions for the third and fourth neighbor Mn pairs are shown in Fig. S13, in units of meV. All other third and fourth neighbor parameters can be obtained through the symmetry operation of magnetic space group $Pa\bar{3}$.

To assess the effect of further-neighbor interactions on the magnon spectrum, we extended the exchange model to include third- and fourth-nearest-neighbor couplings. The corresponding Mn pair geometries are illustrated in Fig. S13, and the calculated interaction parameters are summarized in Table S2.

Among the third- and fourth-neighbor couplings, $J_{3a} = -0.003$ meV and $J_{4a} = -0.005$ meV are negligibly small compared to J_1, whereas $J_{3b} = 0.040$ meV and $J_{4b} = 0.059$ meV are comparable in magnitude to $|J_2| = 0.029$ meV. Their associated DM vectors are either vanishing or at most 0.014 meV, well below $|\vec{D}_1| = 0.063$ meV. As in the case of $\vec{D}_2$, the fourth-neighbor DM interaction $\vec{D}_4$ vanishes by symmetry, since an inversion center is also located at the midpoint of each fourth-neighbor Mn–Mn bond. The inclusion of these interactions shifts the Γ-point magnon energies by approximately 8%, from $(E_4, E_{1\text{-}3}) = (2.40, 3.54)$ meV to $(2.21, 3.57)$ meV.

VII. MAGNON BAND STRUCTURE AND SPIN TEXTURE

In this Supplementary Note, we provide a comprehensive derivation of the magnon eigenstates. Building upon these results, we analyze the magnon energy bands and spin textures presented in Sect. II. B.

1. *Magnon Hamiltonian and its eigenstate*

The effective spin Hamiltonian of $MnTe_2$ is defined by considering both first and second-neighbor exchange interactions:

$$\mathcal{H}_{\text{eff}} = \sum_{\substack{\langle i,j\rangle \\ \alpha,\beta=x,y,z}} J^{(1)}_{ij,\alpha\beta} S^\alpha_i S^\beta_j + \sum_{\substack{\langle\langle i,j\rangle\rangle \\ \alpha,\beta=x,y,z}} J^{(2)}_{ij,\alpha\beta} S^\alpha_i S^\beta_j, \tag{S1}$$

where the spin magnitude is $S = 5/2$, and $J^{(1/2)}_{ij,\alpha\beta}$ represent the anisotropic exchange tensors for the nearest and next-nearest neighbors, respectively. The numerical values of these exchange parameters are determined via density functional theory (DFT) calculations. To analyze the low-energy excitations, we apply the Holstein-Primakoff transformation [S5] within the linear spin-wave approximation. This allows us to express the spin operators S_i in terms of magnon bosonic operators,

$$\begin{bmatrix} \hat{S}^\xi_i \\ \hat{S}^\zeta_i \\ \hat{S}^n_i \end{bmatrix} \approx \begin{bmatrix} \sqrt{S/2}(\hat{a}^\dagger_i + \hat{a}_i) \\ i\sqrt{S/2}(\hat{a}^\dagger_i - \hat{a}_i) \\ S - \hat{a}^\dagger_i \hat{a}_i \end{bmatrix}. \tag{S2}$$

A the local coordinate frame $\{\boldsymbol{\xi}_i, \boldsymbol{\zeta}_i, \boldsymbol{n}_i\}$ is assigned to each Mn atom index $i \in \{1, 2, 3, 4\}$, where the unit vector $\boldsymbol{n}_i$ is aligned with the spin direction specified in Table 1 of the main text. The associated local unit vectors $\boldsymbol{\xi}_i$ and $\boldsymbol{\zeta}_i$ are constructed to satisfy the orthogonality relation $\boldsymbol{n}_i = \boldsymbol{\xi}_i \times \boldsymbol{\zeta}_i$ and $\boldsymbol{\xi}_i \cdot \boldsymbol{\zeta}_i = 0$. The magnon Hamiltonian is then evaluated by transforming the local frames $\{\boldsymbol{\xi}_i, \boldsymbol{\zeta}_i, \boldsymbol{n}_i\}$ into the global Cartesian frame $\{\boldsymbol{x}, \boldsymbol{y}, \boldsymbol{z}\}$ using a rotation matrix R_i. The rotation matrix is given by $(\hat{S}^x_i, \hat{S}^y_i, \hat{S}^z_i)^T = R_i(\hat{S}^\xi_i, \hat{S}^\zeta_i, \hat{S}^n_i)^T$, where ^{T} denotes the transpose.

Applying a Fourier transformation to Eq. (S2) within the linear spin wave regime yields the magnon Hamiltonian $\hat{H} = \sum_{\boldsymbol{k}} \hat{H}_{\boldsymbol{k}} + \mathcal{C}$, where the Hamiltonian in the reciprocal space is expressed as $\hat{H}_{\boldsymbol{k}} = \frac{1}{2}\hat{\Psi}^\dagger_{\boldsymbol{k}} \mathcal{H}_{\boldsymbol{k}} \hat{\Psi}_{\boldsymbol{k}}$ and $\mathcal{C}$ is a constant. The basis of the Hamiltonian matrix $\mathcal{H}_{\boldsymbol{k}}$ is defined as $\hat{\Psi}^\dagger_{\boldsymbol{k}} = ((\hat{a}^1_{\boldsymbol{k}})^\dagger, (\hat{a}^2_{\boldsymbol{k}})^\dagger, (\hat{a}^3_{\boldsymbol{k}})^\dagger, (\hat{a}^4_{\boldsymbol{k}})^\dagger, \hat{a}^1_{-\boldsymbol{k}}, \hat{a}^2_{-\boldsymbol{k}}, \hat{a}^3_{-\boldsymbol{k}}, \hat{a}^4_{-\boldsymbol{k}})$, and † denotes the Hermitian conjugate. We determine the eigenstates of the quadratic magnon Hamiltonian, $|u_{n\boldsymbol{k}}\rangle$, by employing Colpa's procedure [S6] for the diagonalization of bosonic Hamiltonians. Specifically, $|u_{n\boldsymbol{k}}\rangle$ represents a the column vector of the paraunitary eigenvector matrix $U_{\boldsymbol{k}}$ which satisfy the following paraunitary and eigenvalue conditions: $U^\dagger_{\boldsymbol{k}} \eta U_{\boldsymbol{k}} = \eta$, $\eta \mathcal{H}_{\boldsymbol{k}} U_{\boldsymbol{k}} = U_{\boldsymbol{k}} \eta \Lambda_{\boldsymbol{k}}$, and $U^\dagger_{\boldsymbol{k}} \mathcal{H}_{\boldsymbol{k}} U_{\boldsymbol{k}} = \Lambda_{\boldsymbol{k}}$, where $\eta = \sigma_z \otimes I_4$. In these expressions, η is a diagonal matrix defined as $\text{diag}(1, 1, 1, 1, -1, -1, -1, -1)$, and $\Lambda_{\boldsymbol{k}}$ is the diagonal matrix of the energy eigenvalues. These eigenvalues are sorted such that the elements of $\eta\Lambda_{\boldsymbol{k}}$ appear in non-increasing order with decreasing index. The paraunitary eigenvector matrix $U_{\boldsymbol{k}}$ is constructed as $U_{\boldsymbol{k}} = C^{-1}_{\boldsymbol{k}} P^\dagger_{\boldsymbol{k}} \Lambda^{1/2}_{\boldsymbol{k}}$. The matrices $C_{\boldsymbol{k}}$ and $P_{\boldsymbol{k}}$ are obtained from the Cholesky decomposition of the Hamiltonian, $H_{\boldsymbol{k}} = C^\dagger_{\boldsymbol{k}} C_{\boldsymbol{k}}$, and the unitary diagonalization of the resulting product, $C_{\boldsymbol{k}}(\sigma_z \otimes I_4)C^\dagger_{\boldsymbol{k}} = P^\dagger_{\boldsymbol{k}}(\sigma_z \otimes I_4)\Lambda_{\boldsymbol{k}} P_{\boldsymbol{k}}$.

2. *Magnon energy band and spin texture*

By utilizing the obtained eigenstates, we calculate the expectation values of the magnon Hamiltonian and the spin operator, which correspond to the magnon energy bands and magnon spin textures, respectively. The energy bands $E_n(\boldsymbol{k})$ are the (n, n) components of $\Lambda_{\boldsymbol{k}}$ for the band index $n \in \{1, 2, 3, 4\}$. The magnon spin texture is defined as the diagonal components of $U^\dagger_{\boldsymbol{k}} \mathcal{S}_{\boldsymbol{k}} U_{\boldsymbol{k}}$, where the spin operator is given by $\hat{\boldsymbol{S}}_{\boldsymbol{k}} = \frac{1}{2}\hat{\Psi}^\dagger_{\boldsymbol{k}} \mathcal{S}_{\boldsymbol{k}} \hat{\Psi}_{\boldsymbol{k}} = -\sum_i \boldsymbol{n}_i (\hat{a}^i_{\boldsymbol{k}})^\dagger \hat{a}^i_{\boldsymbol{k}}$. For example, the x-component of the spin texture is obtained from the (n, n) components of $U^\dagger_{\boldsymbol{k}} \mathcal{S}^x_{\boldsymbol{k}} U_{\boldsymbol{k}} = U^\dagger_{\boldsymbol{k}}(\mathcal{S}_{\boldsymbol{k}} \cdot \boldsymbol{x})U_{\boldsymbol{k}}$ for the band index $n \in \{1, 2, 3, 4\}$. To distinguish the degenerate points in Fig. S14 (a), we use blue and red coloring on the high-symmetry points and the segments connecting them. The energy bands and the spin textures of the magnon at the non-degenerate points are plotted in Fig. S14 (b–e). In these figures, we can observe their explicit path dependence.

The structure of the spin texture in the reciprocal space, as discussed in the main text, is shown in Fig. S15 (a). Here, we display the magnon spin textures on $k_x = \pm 0.1\pi/a$ and $k_y = \pm 0.1\pi/a$ planes in addition to the $k_z = \pm 0.1\pi/a$

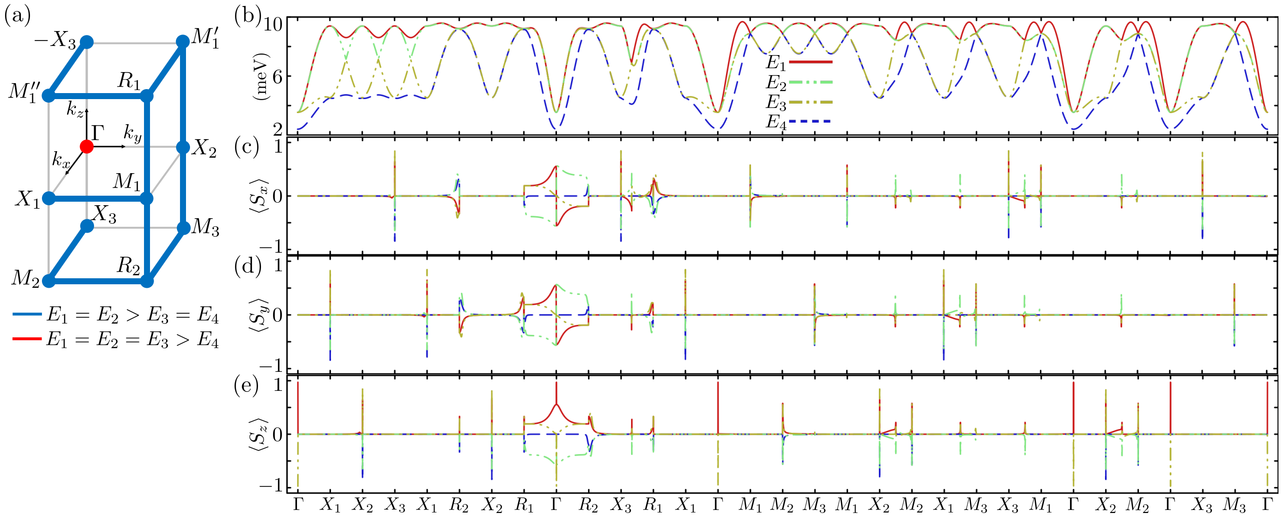

FIG. S14. (a) Schematic representation of the high-symmetry points in the Brillouin zone. (b) Magnon energy bands of the $MnTe_2$ along the path connecting the high-symmetry points, where the bands are non-degenerate along the segments connecting these points. (c–e) The spin-x, y, and z textures of the magnon, respectively, along the path indicated in (b). The spin textures at the high-symmetry points are obtained by choosing the spin-z matrix to be diagonalized, $\mathcal{S}_{\rm diag} = \mathcal{S}_z$.

planes for the $E_{1\text{–}3}$ bands [Fig. S15 (a)] and the E_4 band [Fig. S15 (b)]. Based on the symmetry analysis, we observe that the magnon spin polarization $\langle S_i \rangle$ behaves as an even function under $k_i \to -k_i$ and an odd function under $k_j \to -k_j$, where $j \neq i$, valid for all four bands [Fig. S15 (a, b)] across the Cartesian components $i, j \in \{x, y, z\}$.

At degenerate points, the inherent degree of freedom originating from the degeneracy allows for the mixing of eigenstates constituting the degenerate subspace. This implies that one can choose an arbitrary basis within the degenerate subspace to diagonalize the spin matrix $\boldsymbol{u} \cdot \mathcal{S}_{\boldsymbol{k}}$ for a unit vector $\boldsymbol{u}$, which we denote as $\mathcal{S}_{\rm diag} = \boldsymbol{u} \cdot \mathcal{S}_{\boldsymbol{k}}$, potentially making the spin texture basis-dependent. In this context, the relationship between the magnon spin polarization and crystal momentum, which is observed at non-degenerate points for a single band, is extended to the invariance of the set of magnon spin polarizations of the degenerate bands under that same relation. We will discuss this with examples below.

There are two classes of degenerate points in $MnTe_2$. The first class comprises degenerate points where the set of magnon spin polarizations remains invariant regardless of the choice of the spin matrix $\mathcal{S}_{\rm diag}$. For example, at the high-symmetry points M_1, M_1', M_3, and X_2 in Fig. S16 (b), the set of spin polarizations within the degenerate subspace is invariant under the choice of the spin matrix $\mathcal{S}_{\rm diag}$. Specifically, the sets $\{\langle \boldsymbol{S}_n(\boldsymbol{u}) \rangle | n \in \{1,2\}\}$ and $\{\langle \boldsymbol{S}_n(\boldsymbol{u}) \rangle | n \in \{3,4\}\}$ are invariant under the choice of the spin matrix to be diagonalized, $\mathcal{S}_{\rm diag}$. At the X_1 point, the sets are $\{\langle \boldsymbol{S}_n(\boldsymbol{u}) \rangle | n \in \{1,2\}\} = \{(0, \pm 0.647, 0)\}$ and $\{\langle \boldsymbol{S}_n(\boldsymbol{u}) \rangle | n \in \{3,4\}\} = \{(0, \pm 0.845, 0)\}$. At the M_1 point, both sets yield $\{(\pm 0.577, 0, 0)\}$. Here, the M and X points are invariant under the transformation $k_i \to -k_i$ for $i \in \{x, y, z\}$, and the set of magnon spin polarizations at the M and X points is invariant under flipping the sign of the spin $\langle S_i \rangle \to -\langle S_i \rangle$ and $\langle S_j \rangle \to \langle S_j \rangle$ for $j \neq i$ and $i, j \in \{x, y, z\}$. Thus, the structure of the spins at the M and X points is identical to that at non-degenerate points. Other points, X_2, X_3, M_2, and M_3, show the same feature with different spin direction given by $\bar{3}$ symmetry and the periodicity of the Brillouin zone [Table S3].

M points	$\{\langle \boldsymbol{S}_n(\boldsymbol{u}) \rangle \| n \in \{1,2\}\}$=$\{\langle \boldsymbol{S}_n(\boldsymbol{u}) \rangle \| n \in \{3,4\}\}$	X points	$\{\langle \boldsymbol{S}_n(\boldsymbol{u}) \rangle \| n \in \{1,2\}\}$	$\{\langle \boldsymbol{S}_n(\boldsymbol{u}) \rangle \| n \in \{3,4\}\}$
M_1	$\{(\pm 0.577, 0, 0)\}$	X_1	$\{(0, \pm 0.647, 0)\}$	$\{(0, \pm 0.845, 0)\}$
M_2	$\{(0, 0, \pm 0.577)\}$	X_2	$\{(0, 0, \pm 0.647)\}$	$\{(0, 0, \pm 0.845)\}$
M_3	$\{(0, \pm 0.577, 0)\}$	X_3	$\{(\pm 0.647, 0, 0)\}$	$\{(\pm 0.845, 0, 0)\}$

TABLE S3. Table shows the set of magnon spin polarizations at the M points and X points.

The second class encompasses degenerate points, including the Γ point, the R_1 and R_2 points, and the connecting blue segments [Fig. S14], where the set of spin polarizations varies depending on the choice of the spin matrix $\mathcal{S}_{\rm diag}$. The Γ, R_1, and R_2 points are invariant under the transformation $k_i \to -k_i$ for all $i \in \{x, y, z\}$. At these points, the set of magnon spin polarizations is invariant under simultaneously flipping the sign of a component of the spin polarization and the corresponding component of the spin matrix to be diagonalized, $\mathcal{S}_{\rm diag}$. For instance, at the Γ

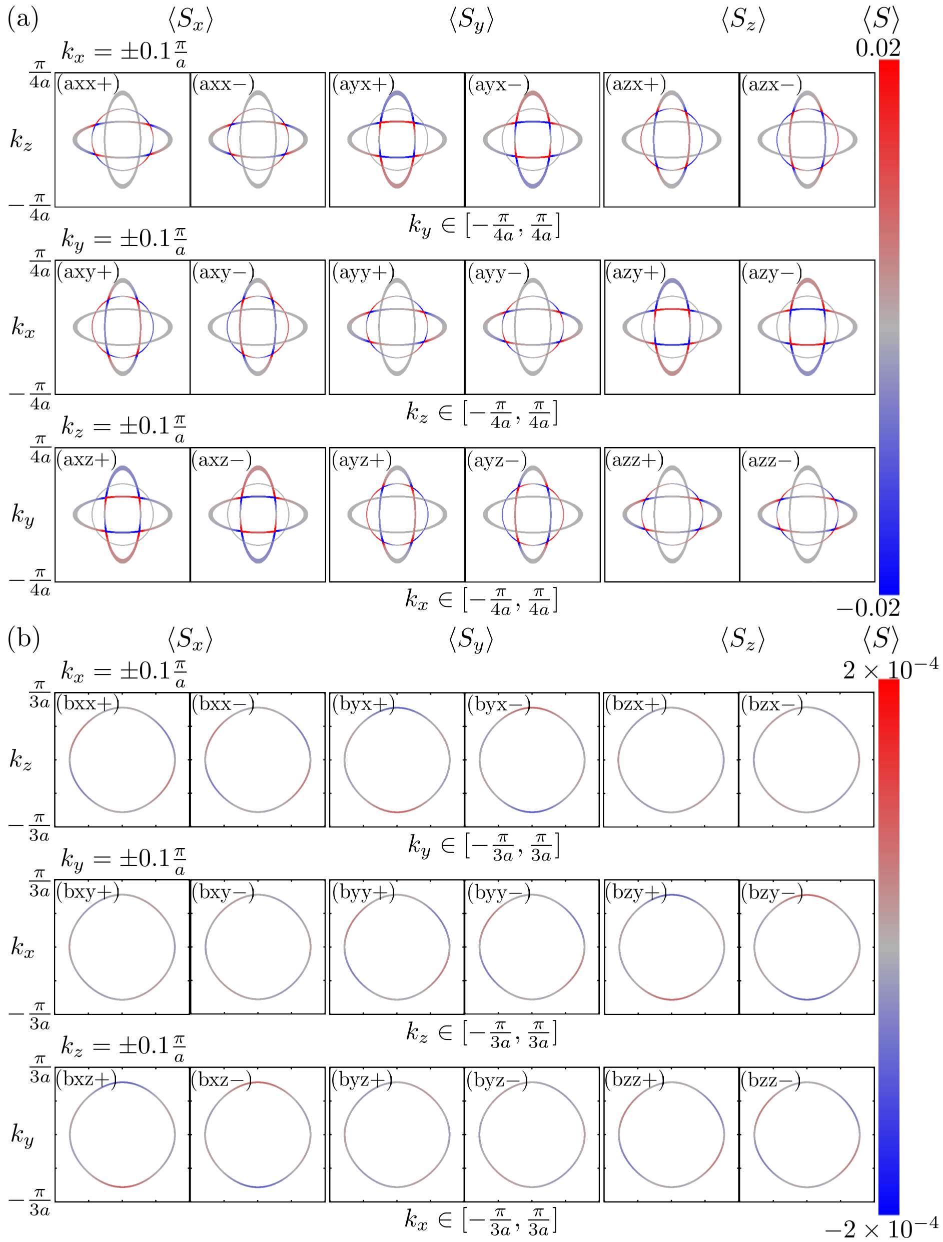

FIG. S15. Panels show spin textures of (a) the $E_{1\text{–}3}$ bands and (b) the E_4 band in the energy windows [3.89 meV, 3.91 meV] and [2.79 meV, 2.81 meV], respectively, on the planes perpendicular to k_x, k_y, and k_z located at $\pm 0.1\pi/a$. We label the spin-i texture of the $E_{1\text{–}3}$ bands on the plane $k_j = \pm 0.1\pi/a$ as (aij±). Similarly, we label the spin textures of (b) the E_4 band.

point, we can diagonalize the spin-z matrix, $\mathcal{S}_{\text{diag}} = \mathcal{S}^z_{\boldsymbol{k}}$, by taking linear combinations of the $E_{1\text{–}3}$ eigenstates. The choice of the spin matrix $\mathcal{S}_{\text{diag}}$ explicitly alters the spin texture, as shown in Fig. S17 (a), where $\mathcal{S}_{\text{diag}} = \mathcal{S}_{\boldsymbol{k}} \cdot \boldsymbol{u}(\theta, \phi)$ with $\boldsymbol{u}(\theta, \phi) = (\sin\theta\cos\phi, \sin\theta\sin\phi, \cos\theta)$ for $\theta \in [0, \pi]$ and $\phi \in [-\pi, \pi)$. Here, the set of magnon spin polarizations is invariant under the transformation $\langle S_i(u_i, u_j)\rangle \to \langle S_i(u_i, -u_j)\rangle$ and $\langle S_j(u_i, u_j)\rangle \to -\langle S_j(u_i, -u_j)\rangle$ for $j \neq i$ where $i, j \in \{x, y, z\}$. The R points also satisfy this relation [Fig. S17 (b)].

Regarding the degenerate points located along the paths between high-symmetry points, the system exhibits broken invariance under $k_i \to -k_i$ for at least one component $i \in \{x, y, z\}$, even if other spatial symmetries are preserved. Specifically, for points on the path R_1-M_1-R_2-M_3-X_2-M_1'-R_1 excluding the high-symmetry points, the set of magnon spin polarizations at the degenerate points is invariant under the relation $\langle S_i(u_i, u_j, k_i, k_j)\rangle \to \langle S_i(u_i, -u_j, -k_i, k_j)\rangle$ and $\langle S_j(u_i, u_j, k_i, k_j)\rangle \to -\langle S_j(u_i, -u_j, -k_i, k_j)\rangle$ for $j \neq i$ where $i, j \in \{x, y, z\}$, which is the general form of the invariance structure of the set of magnon spin polarizations at the degenerate points.

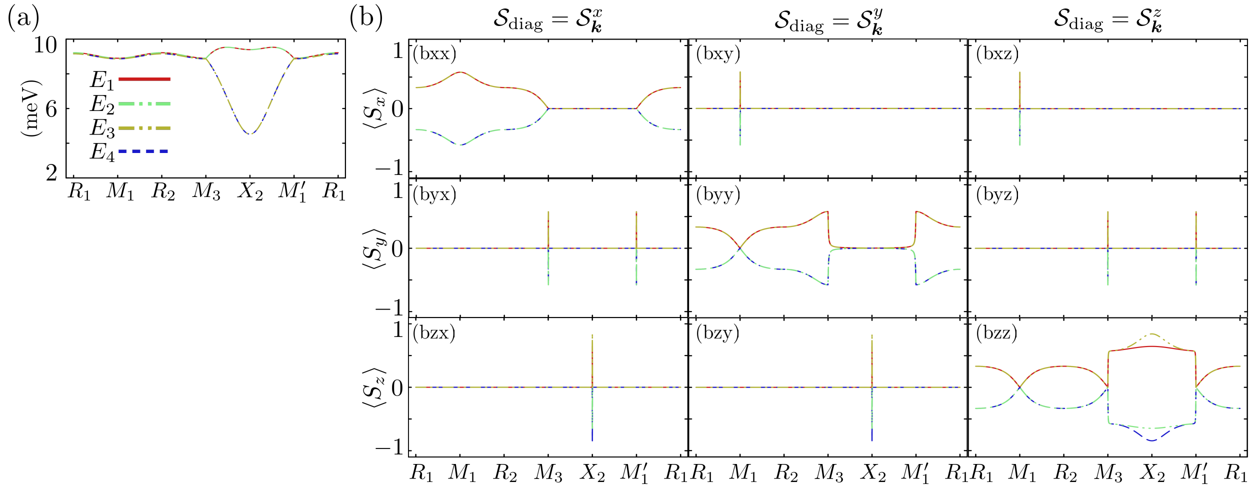

FIG. S16. (a) Magnon energy bands of the $MnTe_2$ along the path connecting the high-symmetry points R_1-M_1-R_2-M_3-X_2-M_1'-R_1 shown in Fig. S14 (a). Here, the bands are degenerate along the whole path. (b) The spin-x, y, and z textures of the magnon with the choices of spin matrices to be diagonalized, $\mathcal{S}_{\rm diag} = \mathcal{S}^x_{\boldsymbol{k}}$, $\mathcal{S}^y_{\boldsymbol{k}}$, and $\mathcal{S}^z_{\boldsymbol{k}}$, along the path connecting the high-symmetry points in (a). We label the spin-i texture of the magnon for $\mathcal{S}_{\rm diag} = \mathcal{S}^j_{\boldsymbol{k}}$ as (bij).

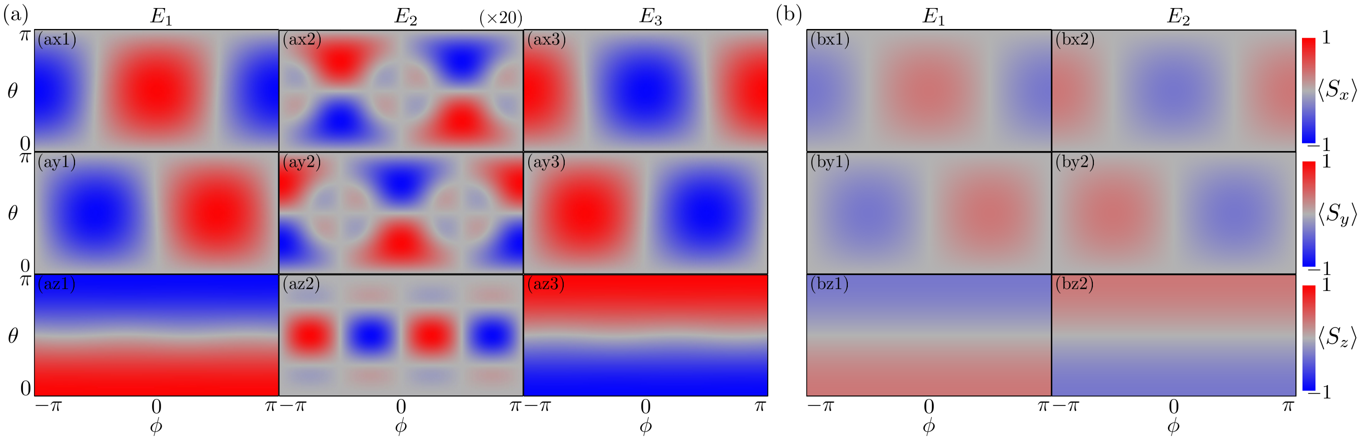

FIG. S17. Magnon spin-x, y, and z polarizations of (a) the triply degenerate bands $E_{1\text{–}3}$ at the Γ point, and (b) the doubly degenerate bands $E_{1\text{–}2}$ at the R point, where the spin polarizations of $E_{3\text{–}4}$ at the R are identical to that of the $E_{1\text{–}2}$, respectively. For each panel, the spin polarization is drawn for the choice of a spin matrix to be diagonalized, $\mathcal{S}_{\rm diag} = \mathcal{S}_{\boldsymbol{k}} \cdot \boldsymbol{u}(\theta,\phi)$, where $\boldsymbol{u}(\theta,\phi) = (\sin\theta\cos\phi, \sin\theta\sin\phi, \cos\theta)$ for $\theta \in [0,\pi]$ and $\phi \in [-\pi,\pi]$. The spin polarization magnitude is magnified by a factor of 20 for (a2) panels. Here, the band indices are used to label the spin polarization; e.g., (x$i m$) indicates the spin-i polarization of the mth band at (x=a) the Γ point or (x=b) the R point.

VIII. ONE-MAGNON RAMAN SCATTERING

Light scattering by a magnon is governed by the magneto-optic coupling in the Fleury-Loudon-Elliott theory[S8, S9]. A coupling Hamiltonian effectively captures the light-matter interaction:

$$\hat{H}_{\rm int} = \epsilon_0 \sum_{\alpha,\beta=x,y,z} E^{\rm sc}_\alpha E^{\rm inc}_\beta \hat{\chi}^{\alpha\beta}, \tag{S3}$$

where $E^{\rm sc}$ and $E^{\rm inc}$ are the scattered and incident electric fields, ϵ_0 is the permittivity of vacuum, and $\chi^{\alpha\beta}$ is the electric susceptibility. The magnetic properties of the system are considered through the susceptibility as a function of spin operators. We can expand the susceptibility in terms of spin operators. Here, we incorporate the Faraday effect and the Cotton-Mouton effect by incorporating the first- and second-order magneto-optical tensors at site i,

$K_{i,\mu}^{\alpha\beta}$ and $G_{i,\mu\nu}^{\alpha\beta}$ [S10, S11]; thus, the susceptibility is written as follows:

$$\hat{\chi}^{\alpha\beta} = \sum_i \left(\sum_{\mu=x,y,z} K_{i,\mu}^{\alpha\beta} \hat{S}_i^{\mu} + \sum_{\mu,\nu=x,y,z} G_{i,\mu\nu}^{\alpha\beta} \hat{S}_i^{\mu} \hat{S}_i^{\nu} \right). \tag{S4}$$

Scattered light polarized along $\boldsymbol{e}_{\rm sc}$ is described by the differential cross section, which is quantified by the correlation of the susceptibility:

$$\frac{d^2\sigma}{d\Omega d\omega_{\rm sc}} = \sum_{\alpha,\beta,\gamma,\rho=x,y,z} e_{\rm sc}^{\alpha} e_{\rm inc}^{\beta} e_{\rm sc}^{\gamma} e_{\rm inc}^{\rho} \mathcal{F} \langle \hat{\chi}^{\alpha\beta}(\boldsymbol{k}) (\hat{\chi}^{\gamma\rho}(\boldsymbol{k}))^\dagger \rangle_{\omega=\omega_{\rm inc}-\omega_{\rm sc}}, \tag{S5}$$

where $\langle \hat{\chi}^{\alpha\beta}(\boldsymbol{k})(\hat{\chi}^{\gamma\rho}(\boldsymbol{k}))^\dagger\rangle_\omega = \int d\omega \int d(\boldsymbol{r'}-\boldsymbol{r''}) e^{-i(\boldsymbol{k}\cdot(\boldsymbol{r'}-\boldsymbol{r''})-\omega t)} \langle \hat{\chi}^{\alpha\beta}(\boldsymbol{r'},t)(\hat{\chi}^{\gamma\rho}(\boldsymbol{r''},0))^\dagger\rangle$, $\omega_{\rm sc}$ and $\omega_{\rm inc}$ are angular frequencies of scattered/incident light, $n_{\rm sc}$ and $n_{\rm inc}$ are refractive indices of medium where scattered/incident light is, c is speed of light, $\mathcal{F} = \omega_{\rm inc}\omega_{\rm sc}^3 n_{\rm sc} \bar{V}/(16\pi^2 c^4 n_{\rm inc})$, and $\bar{V}$ is the scattering volume [S11, S12]. We can relate the correlation function to the Green's function by applying the relation, $\langle \hat{A}\hat{B}\rangle_\omega = -2[n(\omega)+1]{\rm Im}[\langle\langle A;B\rangle\rangle_{\omega+i0^+}]$, where $n(\omega)$ denotes the Bose-Einstein distribution function, 0^+ is an infinitesimal positive number, $\langle\langle A;B\rangle\rangle_\omega$ is a Fourier transformed Green's function defined as $\langle\langle A(t);B(t')\rangle\rangle = -\theta(t-t')\langle[\hat{A}(t),\hat{B}(t')]\rangle$, $\theta(t)$ is the Heaviside function, and $[\cdot,\cdot]$ is the commutator [S13, S14]. Therefore, we compute the Green's function of the spin operators in the susceptibility correlation to obtain the differential scattering cross section. Here, we approximate the correlation using the random phase approximation for the spins at different sites, and using $\langle\langle (\hat{S}_i^z)^2; B\rangle\rangle_\omega = (1-1/2S)\langle S^z\rangle \langle\langle \hat{S}_i^z; B\rangle\rangle_\omega$ for the spins at the same site at the zero-temperature limit [S15, S16]. To compute one-magnon Raman scattering, it is sufficient to consider single-magnon operator correlations after replacing the spin operators with the magnon bosonic operators in Appendix VII. Then, we can define the effective one-magnon susceptibility operator by

$$\hat{\chi}^{(1)\alpha\beta} = \sum_{i,n} \mathcal{X}_{i,n}^{\alpha\beta} \hat{\Psi}_{i,n}, \tag{S6}$$

where $\hat{\Psi}_i^\dagger = ((\hat{a}_i^1)^\dagger, (\hat{a}_i^2)^\dagger, (\hat{a}_i^3)^\dagger, (\hat{a}_i^4)^\dagger, \hat{a}_i^1, \hat{a}_i^2, \hat{a}_i^3, \hat{a}_i^4)$. The susceptibility correlation is reduced to the Green's function of the $\hat{\Psi}$ operator,

$$\langle \hat{\chi}^{(1)\alpha\beta}(\hat{\chi}^{(1)\gamma\rho})^\dagger\rangle_\omega = -2[n(\omega)+1] \sum_{i,j}\sum_{m,n} {\rm Im}\Big[\mathcal{X}_{i,m}^{\alpha\beta}(\mathcal{X}_{j,n}^{\gamma\rho})^* \langle\langle \hat{\Psi}_{i,m}; \hat{\Psi}_{j,n}^\dagger\rangle\rangle_{\omega+i0^+}\Big]. \tag{S7}$$

Since Mn atoms are equivalent, both the first- and second-order magneto-optical tensors are independent of the position index. The susceptibility correlation is simplified to $\sum_{m,n}\mathcal{X}_m^{\alpha\beta}(\mathcal{X}_n^{\gamma\rho})^*\langle\langle \hat{\Psi}_{\boldsymbol{0},m}; \hat{\Psi}_{\boldsymbol{0},n}^\dagger\rangle\rangle_{\omega+i0^+}$, after performing a Fourier transform and setting $\boldsymbol{k}=\boldsymbol{0}$. The Green's function of the magnon operator is derived using its equation of motion,

$$\hbar\omega\langle\langle \hat{\Psi}_{\boldsymbol{0},m}; \hat{\Psi}_{\boldsymbol{0},n}^\dagger\rangle\rangle_\omega = \frac{\hbar}{2\pi}\langle[\hat{\Psi}_{\boldsymbol{0},m}, \hat{\Psi}_{\boldsymbol{0},n}^\dagger]\rangle + \langle\langle [\hat{\Psi}_{\boldsymbol{0},m}, H]; \hat{\Psi}_{\boldsymbol{0},n}^\dagger\rangle\rangle_\omega, \tag{S8}$$

where $\hat{H}$ is the magnon Hamiltonian operator, and $\hbar$ is the reduced Planck constant. Applying the identities $[\hat{\Psi}_{\boldsymbol{0},m}, \hat{\Psi}_{\boldsymbol{0},n}^\dagger] = \eta_{mn}$ and $[\hat{\Psi}_{\boldsymbol{0}}, H] = \eta\hat{\mathcal{H}}_{\boldsymbol{0}}\hat{\Psi}_{\boldsymbol{0}}$ yields $\langle\langle \hat{\Psi}_{\boldsymbol{0},m}; \hat{\Psi}_{\boldsymbol{0},n}^\dagger\rangle\rangle_\omega = \frac{\hbar}{2\pi}\sum_l \left[\hbar\omega - \eta\hat{\mathcal{H}}_{\boldsymbol{0}}\right]_{ml}^{-1}\eta_{ln}$. Performing the Bogoliubov-de Gennes transformation, we obtain the differential cross section of one-magnon Raman scattering as follows:

$$\frac{d^2\sigma}{d\Omega d\omega_{\rm sc}} = -\sum_{\alpha,\beta,\gamma,\rho=x,y,z} e_{\rm sc}^{\alpha} e_{\rm inc}^{\beta} e_{\rm sc}^{\gamma} e_{\rm inc}^{\rho} \frac{\hbar}{\pi}\mathcal{F}[n(\omega)+1] \sum_{n,m} {\rm Im}\Big[\mathcal{X}_m^{\alpha\beta}(\mathcal{X}_n^{\gamma\rho})^*\left[U_{\boldsymbol{0}}(\omega\eta + i\eta 0^+ - \Lambda_{\boldsymbol{0}})^{-1}U_{\boldsymbol{0}}^\dagger\right]_{mn}\Big]\Bigg|_{\omega=\omega_{\rm inc}-\omega_{\rm sc}}. \tag{S9}$$

Using the Sokhotski-Plemelj identity, $(\omega \pm i0^+)^{-1} = \mathcal{P}\frac{1}{\omega} \mp i\pi\delta(\omega)$, where $\mathcal{P}$ denotes the Cauchy principal value, the integrated scattering intensity, $d\sigma/d\Omega$, is derived by integrating over $\omega_{\rm sc}$.

We investigated the one-magnon Raman scattering using the first- and second-order magneto-optical tensors, $K_\mu^{\alpha\beta}$ and $G_{\mu\nu}^{\alpha\beta}$. Due to the symmetry of $MnTe_2$, first- and second-order magneto-optical tensors are simplified as follows:

$$\begin{aligned} &K_z^{xy} = K_x^{yz} = K_y^{zx} = -K_z^{yx} = -K_x^{zy} = -K_y^{xz} = iF_{123}, \\ &G_{xx}^{xx} = G_{yy}^{yy} = G_{zz}^{zz}, \quad G_{zz}^{xx} = G_{xx}^{yy} = G_{yy}^{zz}, \quad G_{yy}^{xx} = G_{zz}^{yy} = G_{xx}^{zz}, \\ &G_{xy}^{xy} = G_{yx}^{xy} = G_{xy}^{yx} = G_{yx}^{yx} = G_{xz}^{xz} = G_{zx}^{xz} = G_{xz}^{zx} = G_{zx}^{zx} = G_{yz}^{yz} = G_{zy}^{yz} = G_{yz}^{zy} = G_{zy}^{zy}, \end{aligned} \tag{S10}$$

and other components of $K_\mu^{\alpha\beta}$ and $G_{\mu\nu}^{\alpha\beta}$ are zero [S7]. Here, F_{123} and the components of the second-order magneto-optical tensor are real numbers. From this calculation, the RL/LR chiral imbalance is rooted in the coupling between first- and second-order magneto-optic interactions.

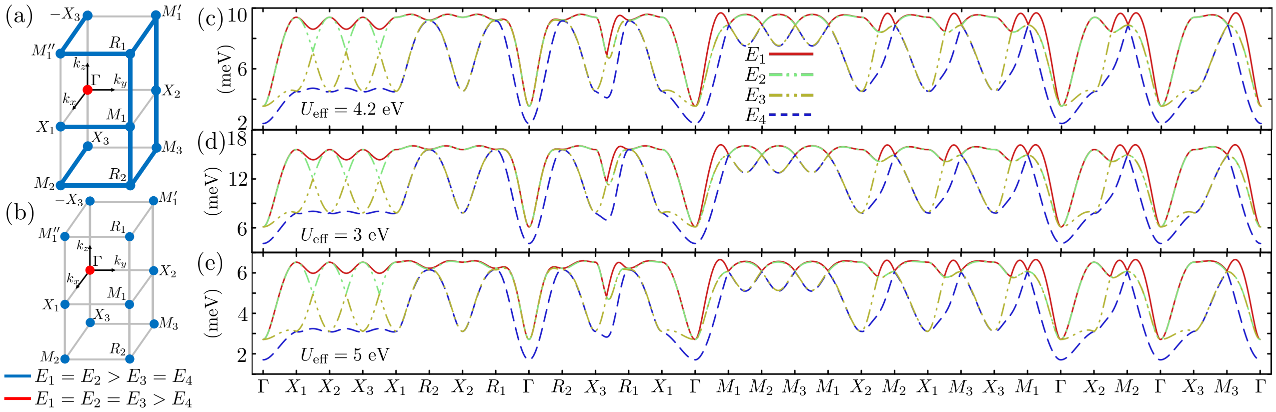

FIG. S18. Schematic representation of the high-symmetry points in the Brillouin zone for (a) $U_{\rm eff} = 4.2$ eV and (b) $U_{\rm eff} = 3$ eV and 5 eV, where the degenerate points and lines are colored blue and red. (c–e) Magnon energy bands of the $MnTe_2$ along the path connecting the high-symmetry points for (c) $U_{\rm eff} = 4.2$ eV, (d) $U_{\rm eff} = 3$ eV, and (e) $U_{\rm eff} = 5$ eV.

IX. $U_{\rm eff}$ DEPENDENCE OF EXCHANGE PARAMETERS AND MAGNON BANDS

To systematically assess the sensitivity of the magnetic exchange parameters to the choice of on-site correlation, we performed DFT+U calculations at $U_{\rm eff} = 3.0$ and 5.0 eV in addition to the main value of $U_{\rm eff} = 4.2$ eV. The resulting first- and second-neighbor interaction parameters are summarized in Tables S4 and S5, and the corresponding magnon band structures are shown in Fig. S18.

At $U_{\rm eff} = 3.0$ eV, the nearest-neighbor coupling increases to $J_1 = 1.094$ meV with $|\vec{D}_1| = 0.106$ meV, while the second-neighbor exchange strengthens to $J_2 = -0.064$ meV. At $U_{\rm eff} = 5.0$ eV, the couplings decrease to $J_1 = 0.470$ meV, $|\vec{D}_1| = 0.045$ meV, and $J_2 = -0.011$ meV. Although the absolute magnitudes of J_1 and $|\vec{D}_1|$ vary by more than a factor of two across this range, the ratio $|\vec{D}_1|/J_1$ remains remarkably stable: 0.097 ($U_{\rm eff} = 3.0$ eV), 0.093 ($U_{\rm eff} = 4.2$ eV), and 0.096 ($U_{\rm eff} = 5.0$ eV). The degeneracies of the magnon bands at the high-symmetry points [red and blue points in Fig. S18 (a–b)] are preserved in this range, while the degeneracy connecting them—blue lines in Fig. S18 (a)—is lifted. In contrast to the degeneracy at the high-symmetry points, the magnon energies at the Γ-point decrease from $(E_4, E_{1\text{-}3}) = (4.08, 6.12)$ meV to $(1.70, 2.71)$ meV as $U_{\rm eff}$ increases from 3 eV to 5 eV. Since the experiment investigates magnons at the Γ-point, our choice of $U_{\rm eff}$ accurately describes the experimental magnon energy with threefold degeneracy.

TABLE S4. Calculated first- and second-neighbor magnetic interaction parameters in units of meV for $U_{\rm eff} = 3$ eV. Here, with Mn_1(0, 0, 0) as the reference, $n = 1$ and $n = 2$ correspond to its pairs with Mn_2(-0.5, 0.5, 0) and Mn_1(0, 0, 1), respectively. All other symmetry-equivalent interactions are obtained via the magnetic space group $Pa\bar{3}$.

	J_n	D_n^x	D_n^y	D_n^z	Γ_n^{xx}	Γ_n^{yy}	Γ_n^{zz}	Γ_n^{xy}	Γ_n^{yz}	Γ_n^{zx}
$n = 1$	1.094	0.000	−0.050	0.093	0.002	−0.002	0.000	−0.005	−0.005	0.002
$n = 2$	−0.064	0.000	0.000	0.000	−0.003	0.006	−0.003	0.014	0.000	−0.003

TABLE S5. Calculated first- and second-neighbor magnetic interaction parameters in units of meV for $U_{\rm eff} = 5$ eV. Here, with Mn_1(0, 0, 0) as the reference, $n = 1$ and $n = 2$ correspond to its pairs with Mn_2(-0.5, 0.5, 0) and Mn_1(0, 0, 1), respectively. All other symmetry-equivalent interactions are obtained via the magnetic space group $Pa\bar{3}$.

	J_n	D_n^x	D_n^y	D_n^z	Γ_n^{xx}	Γ_n^{yy}	Γ_n^{zz}	Γ_n^{xy}	Γ_n^{yz}	Γ_n^{zx}
$n = 1$	0.470	0.006	−0.014	0.042	0.000	0.000	0.000	−0.003	0.000	0.000
$n = 2$	−0.011	0.000	0.000	0.000	−0.002	0.003	−0.002	0.005	0.000	−0.002

[S1] I. Oftedal, Zeitschrift für Physikalische Chemie **135**, 291 (1928).
[S2] O. Okada and T. Miyadai, Magnetic susceptibility of $MnTe_2$ with pyrite structure. J. Phys. Soc. Jpn. **43**, 343 (1977).
[S3] A. Milosavljević, A. Šolajić, J. Pešić, Yu Liu, C. Petrovic, N. Lazarević, and Z. V. Popović, Evidence of spin-phonon coupling in $CrSiTe_3$. Phys. Rev. B **98**, 104306 (2018).
[S4] M. J. Gray, N. Kumar, R. O'Connor, M. Hoek, E. Sheridan, M. C. Doyle, M. L. Romanelli, G. B. Osterhoudt, Y. Wang, V. Plisson, S. Lei, R. Zhong, B. Rachmilowitz, H. Zhao, H. Kitadai, S. Shepard, L. M. Schoop, G. D. Gu, I. Zeljkovic, X. Ling, and K. S. Burch, A cleanroom in a glovebox. Rev. Sci. Instrum. **91**, 073909 (2020).
[S5] T. Holstein, H. Primakoff, Field Dependence of the Intrinsic Domain Magnetization of a Ferromagnet, Phys. Rev. **58**, 1098 (1940).
[S6] J. H. P. Colpa, Diagonalization of the quadratic boson hamiltonian, Physica A **93**, 327 (1978).
[S7] S. V. Gallego, J. Etxebaria, L. Elcoro, E. S. Tasci and J. M. Perez-Mato, Automatic calculation on symmetry-adapted tensors in magnetic and non-magnetic materials: new tool of the Bilbao Crystallographic Server, Acta Cryst. **A75**, 438-447 (2019).
[S8] P. A. Fleury, R. Loudon, Scattering of Light by One- and Two-Magnon Excitations, Phys. Rev. **166**, 514 (1968).
[S9] R. J. Elliott, R. Loudon, Theory of the absorption edge in semiconductors in a high magnetic field, J. Phys. Chem. Solids **15**, 196 (1960).
[S10] Yu. G. Pashkevich, S. A. Fedorov, A. V. Eremenko, V. L. Sobolev, One-magnon light scattering in exchange-noncollinear magnets, Ferroelectrics **162**, 237 (1994).
[S11] M. G. Cottam, D. J. Lockwood, *Light Scattering in Magnetic Solids* (John Wiley & Sons, New York, 1986).
[S12] M. G. Cottam, On the temperature dependence of one-magnon light scattering in antiferromagnets, J. Phys. C: Solid State Phys. **8**, 1933 (1975).
[S13] D. N. Zubarev, Double-time Green functions in statistical physics, Sov. Phys. Usp. **3** 320 (1960).
[S14] N. Majlis, *The Quantum Theory of Magnetism*, 2nd ed. (World Scientific, Singapore, 2007).
[S15] F. B. Anderson, H. B. Callen, Statistical Mechanics and Field-Induced Phase Transitions of the Heisenberg Antiferromagnet, Phys. Rev. **136**, A1068, (1964).
[S16] M. E. Lines, Sensitivity of Curie Temperature to Crystal-Field Anisotropy. I. Theory, Phys. Rev. **156**, 534 (1967).
